# Phenotypic plasticity of life-history traits of a calanoid copepod in a tropical lake: Is the magnitude of thermal plasticity related to thermal variability?

**DOI:** 10.1371/journal.pone.0196496

**Published:** 2018-04-30

**Authors:** Elizabeth Ortega-Mayagoitia, Osvaldo Hernández-Martínez, Jorge Ciros-Pérez

**Affiliations:** 1 Grupo de Investigación en Limnología Tropical, División de Investigación y Posgrado, Facultad de Estudios Superiores Iztacala, Universidad Nacional Autónoma de México, Tlalnepantla de Baz, Edo, de México, México; 2 Posgrado en Ciencias del Mar y Limnología, Universidad Nacional Autónoma de México, Tlalnepantla de Baz, Edo, de México, México; Universidad de la Republica Uruguay, URUGUAY

## Abstract

According to the Climatic Variability Hypothesis [CVH], thermal plasticity should be wider in organisms from temperate environments, but is unlikely to occur in tropical latitudes where temperature fluctuations are narrow. In copepods, food availability has been suggested as the main driver of phenotypic variability in adult size if the range of temperature change is less than 14°C. *Leptodiaptomus garciai* is a calanoid copepod inhabiting Lake Alchichica, a monomictic, tropical lake in Mexico that experiences regular, narrow temperature fluctuations but wide changes in phytoplankton availability. We investigated whether the seasonal fluctuations of temperature and food produce phenotypic variation in the life-history traits of this tropical species. We sampled *L*. *garciai* throughout a year and measured female size, egg size and number, and hatching success, along with temperature and phytoplankton biomass. The amplitude of the plastic responses was estimated with the Phenotypic Plasticity Index. This index was also computed for a published dataset of 84 copepod populations to look if there is a relationship between the amplitude of the phenotypic plasticity of adult size and seasonal change in temperature. The temperature annual range in Lake Alchichica was 3.2°C, whereas phytoplankton abundance varied 17-fold. A strong pattern of thermal plasticity in egg size and adult female size followed the inverse relationship with temperature commonly observed in temperate environments, although its adaptive value was not demonstrated. Egg number, relative reproductive effort and number of nauplii per female were clearly plastic to food availability, allowing organisms to increase their fitness. When comparing copepod species from different latitudes, we found that the magnitude of thermal plasticity of adult size is not related to the range of temperature variation; furthermore, thermal plasticity exists even in environments of limited temperature variation, where the response is more intense compared to temperate populations.

## Introduction

Phenotypic plasticity, the ability of an organism to respond to environmental stimuli by changing its phenotype [1] has been recorded for diverse species, response traits and environmental variables. The investigative and interpretative approaches are multifaceted, and sometimes are controversial, because whereas many authors argue that the concept of phenotypic plasticity should be restricted to developmental processes, others include more easily changing traits as physiology and behaviour [2]. Temperature is one of the abiotic factors most extensively studied regarding phenotypic variability, and some patterns of thermal plasticity in ectotherms are well known [3]. For example, in many species the size of adults is inversely related to the temperature of the environment where they developed; this, the temperature–size rule (TSR), affects other life-history traits such as size and number of descendants [4]. However, it is not known whether thermal plasticity of body size in ectotherms is adaptive [4], and its physiological mechanisms remain unclear [3,5–7].

In tropical environments, the effects of temperature on life-history traits of aquatic invertebrates have received scant attention, perhaps because seasonal fluctuations in the tropics have been considered too small to exert relevant ecological and evolutionary effects. In tropical seas, annual temperature fluctuations are about 2–5°C [8], whereas fluctuations of 20°C or higher are common in temperate coastal zones [9–11]. According to the Climatic Variability Hypothesis, natural selection would favour adaptive traits allowing organisms to withstand the contrasting and challenging conditions of fluctuating temperate environments [12,13]. Among those traits, the capacity to produce alternative phenotypes in response to the thermal variability (and the accompanying changes in biotic and abiotic conditions) would be advantageous. In contrast, tropical organisms, experiencing narrow temperature ranges, are not in need of differentiated phenotypes, and phenotypic plasticity would be weak or absent [14–16].

Seasonal variations in female size and reproductive traits of copepods in temperate latitudes have often been attributed to temperature changes, even though thermal plasticity is not explicitly stated [9–11,17]. Copepods, like many other ectotherms, usually follow the TSR: adults reach larger sizes when they develop at low water temperature and vice versa [7,18]. However, there are many exceptions, and opposing responses in adult size and reproductive traits to temperature have been observed even between species of the same genus [19,20]. In addition, it has been demonstrated experimentally that traits in some species may also be strongly affected by food availability, alone or in combination with temperature [19].

A multi-species study at a Mediterranean location suggested that a temporal variation of at least 14°C was necessary for temperature to override the influence of food on adult copepod size [11]; accordingly, in tropical environments food should be the main driver of adult size. In a recent, extensive meta-analysis of the seasonal trends in adult size of subtropical and temperate copepods [21], the TSR was observed in 42 of 48 species, suggesting that temperature, not food (measured as chlorophyll *a* concentration), most consistently explained variation in adult body size. No relationship was found between the strength or direction of the thermal response and latitude; regrettably, the lack of suitable information from tropical environments precluded comparison of thermal responses along a broader latitudinal gradient. It is remarkable that although many hydrobiological investigations monitor the abundance of animal populations, the size of the individuals is not always recorded.

*Leptodiaptomus garciai* (Osorio-Tafall, 1942) is a microendemic calanoid copepod that exclusively inhabits Lake Alchichica, a warm-monomictic, tropical lake with regular, narrow oscillations in temperature but with wide changes in resource availability throughout the year; this constitutes an ideal scenario to test the effects of temperature and food on the size of a tropical ectothermic species. Fragmented information on adult size hinted at wide intra-annual variability, but a reliable data set was lacking. Accordingly, to determine whether seasonal fluctuations in temperature and/or food can produce phenotypic variation in the life-history traits of this tropical species, we investigated the following: 1) the extent of phenotypic variation in adult female size and reproductive traits of *L*. *garciai*; 2) whether such plasticity can be related to temporal variability of temperature and/or resource (phytoplankton) availability; 3) the trade-offs among life-history traits involved. We sampled *L*. *garciai* throughout a year and measured female size, egg size and number, and hatching success. At the same time, we measured the temperature of the water column and assessed phytoplankton biomass. In addition, we examined data from the literature to 4) test the climatic variability hypothesis, looking for a relationship between the amplitude of phenotypic plasticity in the size of adult copepod females and several indicators of temperature variability.

## Methods

### Ethics statement

We collected copepods in a lake that is public property, but not under protection by Mexican laws. Further, zooplankton is not under any protection law and *L*. *garciai* is not an endangered species; thus, no specific permissions were required to collect samples.

Lake Alchichica is a saline crater lake (Total Dissolved Solids, TDS, 8.9 g L^-1^; specific conductivity at 25°C, K_25_ = 12940 μS cm^-1^) within a cluster of 8 lakes with distinct salinities located in the Cuenca Oriental, a high-altitude plateau in central Mexico (19°22’N, 97°24’W; 2300 m a.s.l.). This waterbody is warm-monomictic; the water column circulates in winter (December or January to March) and remains stratified the rest of the year. The maximum depth of this lake is 63 m and during the stratification period the hypolimnion becomes completely anoxic. The lowest average temperature of the mixing layer is quite stable (14.8 ± 0.1°C during the winter mixing of the water column) but the highest average temperature varies among years. Adame et al. [22] reported a mean of 17.2 ± 1.4°C for epilimnetic waters from 1999 to 2002, but Ardiles et al. [23] observed 19.9 ± 0.7°C in 2007. Hence, the temperature annual range (TAR; i.e., the difference between the lowest–in winter- and the highest–in summer- average temperature at the mixed, oxygenated layer) may oscillate from 2.8 to about 5.0°C.

Owing to its nutrient concentrations and primary production, Lake Alchichica is oligotrophic [22], but this condition fluctuates seasonally (see the Results section). The large central diatom *Cyclotella alchichicana* [24] (average diameter 50 μm) constitutes the main component of phytoplanktonic biomass, forming a winter bloom during the mixing of the water column and a deep chlorophyll maximum (DCM) that develops at the metalimnion during the stratification. Other common phytoplanktonic species are the diatoms *Cyclotella choctawhatcheeana* and *Chaetoceros muelleri*, the chlorophytes *Monoraphidium minutum*, *Oocystis parva* and *Oocystis submarina*, and the cyanobacteria *Nodularia spumigena* and *Synechocystis aquatilis* [25,26]. The copepod *L*. *garciai* is the main component of the zooplankton assemblage; in a previous paper we concluded that it is present throughout the year despite the often-limiting availability of phytoplankton, owing to its low food threshold (C_0_) and high resistance to starvation; in addition, it performs diel vertical migrations to avoid light-related mortality factors (UV damage and/or predation risk) [27]. There are also two rotifer species (*Brachionus* sp. ‘Mexico’ and *Hexarthra jenkinae*) and a cyclopoid copepod (*Euclyclops* cf. *pectinifer*) that contribute minor quantities of biomass [26]. The silverside *Poblana alchichica* is the only zooplanktivorous predator in the lake [28].

Sampling was performed monthly from January 2008 to January 2009 at a single spot in the central, deepest zone of the lake, at midday. We measured temperature and dissolved oxygen each meter throughout the water column with a Hydrolab Datasonde 3/Surveyor 3 multiparameter water quality logging system. As we intend to describe the environmental conditions experienced by copepods at a given day, we took into account that adult individuals of *L*. *garciai* perform diel vertical migrations–moving across a vertical gradient of biotic and abiotic conditions- and avoid the anoxic hypolimnion during the summer stratification of the lake [27]. In addition, we assumed they also evade the surface of the water column as has been observed in crustacean zooplankton inhabiting other highly transparent lakes [29]. Thus, for each month we calculated the arithmetic mean of the temperature measurements at the epilimnion plus the metalimnion (*n* = 26 to 63), excluding the upper 1 m of the epilimnion––and the anoxic layer, when present. The year this study was performed, there was oxygen throughout the water column from January to March; the anoxic layer was first detected in April at 57 m at the onset of the stratification period; then gradually became wider and reached its maximum expansion in September at 27 m; from that moment onwards, the anoxic layer contracted to the bottom and in December it was observed at 55 m (see S1 Dataset file). Detailed long-term descriptions of the hydrodynamics of Lake Alchichica have been published elsewhere [30].

Phytoplankton is evenly distributed in the water column during the mixing period and forms a deep chlorophyll maximum at the metalimnion (DCM) during the stratification [26, 27]. We obtained phytoplankton samples with a Niskin-type bottle (6 L) at 10 and 25 m when the water column was mixing and at the epilimnion (8.5 m) and the DCM when the lake was stratified. 500 mL of the sample were fixed with Lugol’s solution. Phytoplankton was counted by the Utermöhl method [31], counting at least 100 individuals of the most abundant species at ×200, ×400 and ×1000 magnifications, according to the size of organisms. Phytoplankton biovolume was calculated by means of geometric formulae [32] and was transformed to biomass assuming a cellular density of 1 (1 cm^3^ = 1 g).

Copepods were obtained from vertical net hauls (pore size 120 μm) from a depth of 40 m from December to June or from the top of the anoxic layer (between 36 and 26 m) during the stratification. Organisms for size measurements were fixed with formaldehyde diluted with filtered lake water (final concentration 4%). A share of the net haul was kept alive and transported to the laboratory to monitor hatching success. Each month, we measured the total length (excluding furcal rami) of 50 ovigerous females with a Leica DMIL inverted microscope at ×400. The dry weight of individuals was calculated from the length-weight model [33]:

$$W=7.9\times 10^{-7}L^{2.33},$$

where *W* is the dry weight in μg and *L* is the length of the individual in μm. Further, we estimated the wet weight as dry weight ×10 [34]. This procedure allows having all zooplankton and phytoplankton biomass calculations expressed in the same units.

Reproduction in *L*. *garciai* is typical of diaptomids: after mating, females produce a single egg-mass (the sac) that remains attached to the urosome until all eggs have hatched; re-mating is necessary to produce a new clutch [35,36]. In each of 100 females, clutch size (number of eggs per sac) was recorded and eggs were measured at ×400 to calculate their volume from geometric formulae [sphere or prolate spheroid]. Assuming a density of 1 (1 cm^3^ = 1 g), volume was transformed to wet weight. Reproductive effort (RE), defined as the total biomass invested by a female in a single clutch, was calculated according to Caley, Schwarzkopf and Shine [37]:

RE=EW×CS

where *EW* is egg weight and *CS* is clutch size.

To assess hatching success, each month we isolated 40–60 ovigerous females (from the vertical haul) which were placed individually in eight-well Evergreen™ culture plates with 8 mL of filtered epilimnetic water. Plates were placed on an orbital platform shaker at 40 rpm in a temperature-controlled room at 18 ± 1°C, and 12:12 light: dark cycle. Clutch size was recorded and plates were monitored every 24 hours to document hatching of nauplii. Females were transferred daily to fresh medium until all eggs in the sac hatched or showed signs of decomposition. Raw data of life-history traits of *L*. *garciai* can be consulted in the S1 Dataset file in Supporting information.

The assessment of phenotypic plasticity in *L*. *garciai* involved the comparison of population means of the life-history traits throughout the corresponding conditions of temperature and food observed in the field. This approach allows addressing ecological questions where the focus is the response of the population to environmental gradients [38]. For this purpose, all life-history traits measured individually and data of vertical profiles of temperature and phytoplankton from a given sampling date were averaged. The population means of every life-history trait (phenotypes) were plotted against the averages of temperature and food (environments) to obtain the population reaction norms. The numerical relationships among phenotypes and environment were explored with Pearson’s correlation tests. Data were transformed in case they did not meet normality or variance requirements, or a Spearman rank-order correlation test was performed. We considered that a given life-history trait had a mean plastic response if the mean reaction norm had a significantly non-zero slope [39]. To estimate the amplitude of the plastic responses, we calculated the Phenotypic Plasticity Index (PPI) [40], which is a ratio based on the maximum and minimum population averages observed throughout the study: ([maximum mean–minimum mean]/maximum mean); it was multiplied by 100 to express it as a percentage of change. We refer to the PPI result as the absolute plasticity of a given trait, and we used it to test whether temperature or food availability determines the amplitude of the phenotypic plasticity for adult size and reproductive traits. Relationships between traits were surveyed with Pearson’s or Spearman rank order correlation tests. Negative functional relationships among traits were considered trade-offs [41]. In addition, to evaluate if a given life-history trait was simultaneously influenced by temperature and phytoplankton abundance, we used generalized additive models (GAMs) fit in R v. 3.4.2. [42] using the *gam* package [43, 44]. Further details of this procedure can be found in S1 Table.

To analyse our results within a global perspective, we downloaded the database generously made public by Horne *et al*. [21]; this database is a compilation of published information of temporal variation in copepod body size (both sexes, reported as dry biomass) and the corresponding water temperature, plus geographical location. From the dataseries of each population, we extracted the maximum and minimum monthly average body size of adult females to calculate the PPI as previously described. We excluded two populations that had data from only three or four consecutive months (i.e., *Paracalanus parvus* from Marshall, 1949, and *Parvocalanus crassirostris* from Sun et al., 2012, respectively), the remaining 80 copepod populations belong to 46 species of the orders Calanoida, Cyclopoida, Harpacticoida, and Poecilostomatoida. Into the global comparison, we incorporated data from our study (*L*. *garciai*) plus data of two tropical populations of the freshwater *Thermocyclops minutus* [45], one of the tropical population of the marine *Acrocalanus giber* [46], and another of the temperate *Epischura baicalensis* [47]; this gave a total of 85 populations from 50 species. The added papers reported size and temperature data from a whole seasonal cycle, embracing a significant portion of the water column. Length of females was transformed to biomass using the nearest length-weight models available [21,33]. We analysed data at population and species level separately. For the second one, we selected the population with the highest PPI. As explicative variables of phenotypic plasticity, we considered: 1) latitude, 2) temperature of the coldest month (*T*_min_), 3) temperature of the warmest month (*T*_max_), 3) temperature annual range (i.e., TAR, *T*_max_−*T*_min_), 4) temperature seasonality (standard deviation of the monthly temperature). We used the temperature annual range to calculate the % of change in biomass per °C as a proxy for the intensity of phenotypic plasticity (PPI/TAR). Spearman correlations were performed with Sigma Plot for Windows Version 11.0 (Systat Software Inc., 2008).

**Fig 1:**
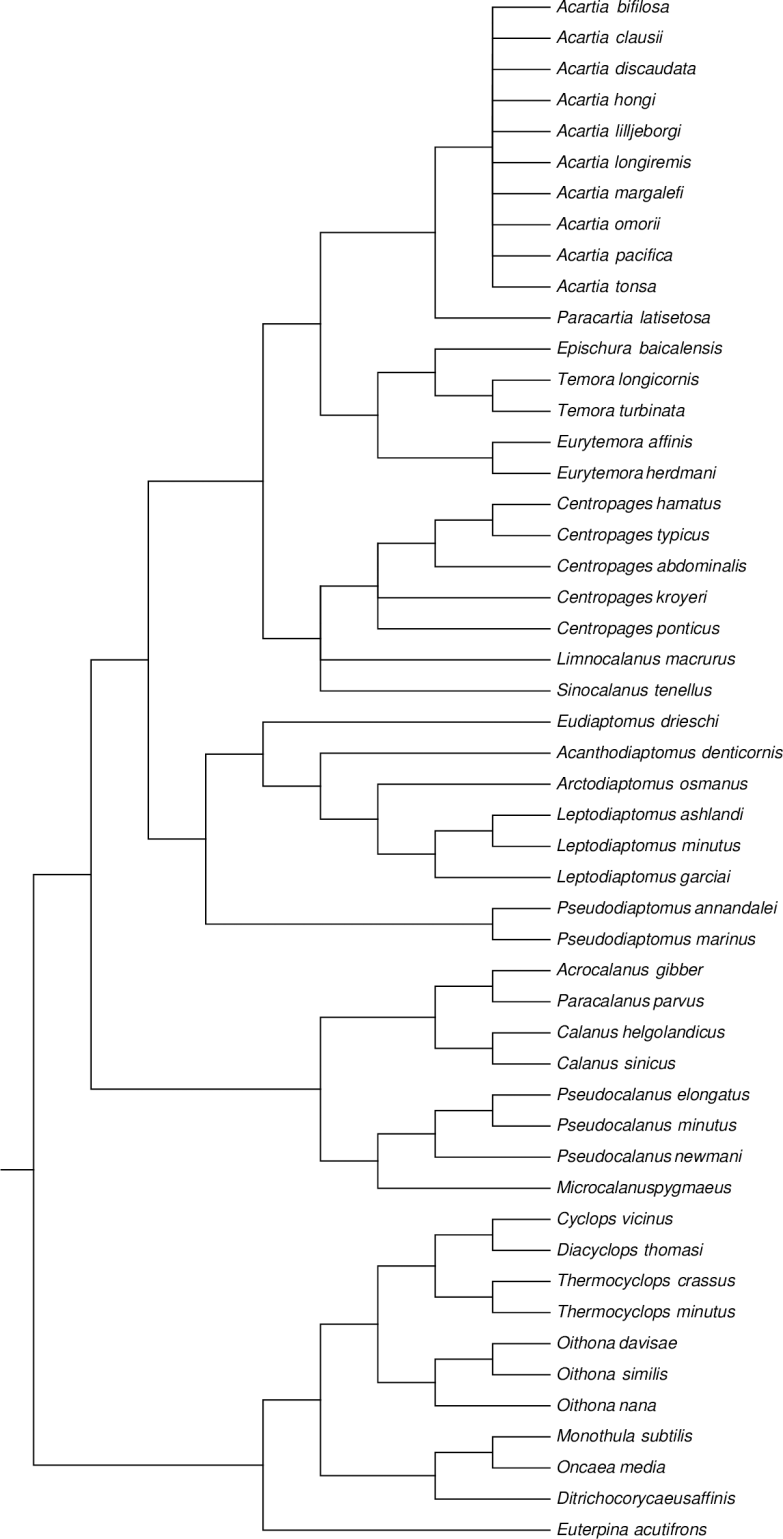
Phylogenetic tree of the 85 copepod species included in the global analysis of phenotypic plasticity of female size. This phylogeny was assembled using diverse sources and was used to perform phylogenetically informed contrasts.

Additionally, we performed phylogenetic comparative contrasts (PICs, sensu Felsenstein [48]) to consider the possible effect of phylogeny on the phenotypic plasticity (PPI) of the analysed copepods. As the database comprises three copepod Orders it was necessary to assemble a phylogeny using diverse sources (Fig 1). The relationships between Orders and the arrangement of families within Orders followed Khodami et al. [49]. For families with more than two species we used published phylogenetic trees: Centropagidae [50], Diaptomidae [51], Paracalanidae [52], Clausocalanidae [53], Cyclopidae [54] and Oithonidae [55]. The phylogeny of *Acartia* species remains unsolved [56] so all the species within the genus were left as soft polytomies; Acartidae was placed as a sister family of Temoridae [57]. Temoridae was resolved using COI sequences downloaded from GenBank (https://www.ncbi.nlm.nih.gov/genbank/) (accession numbers: JX995146.1, KF977353.1, KC683830.1, HM473958.1, CAISN1513-14). The phylogeny of Temoridae was inferred using Maximum Likelihood with a GTR model and 1000 bootstrap replicates in the software MEGA6 [58]. PICs for the species dataset were performed using the *crunch* algorithm within the software Comparative Analyses of Phylogenetics and Evolution in R version 0.5.2 [59]. The internal branch length for calculating contrasts at polytomies following Pagel’s method [60] was set to 1, whereas for the other branches the length was set to 2.

## Results

**Fig 2:**
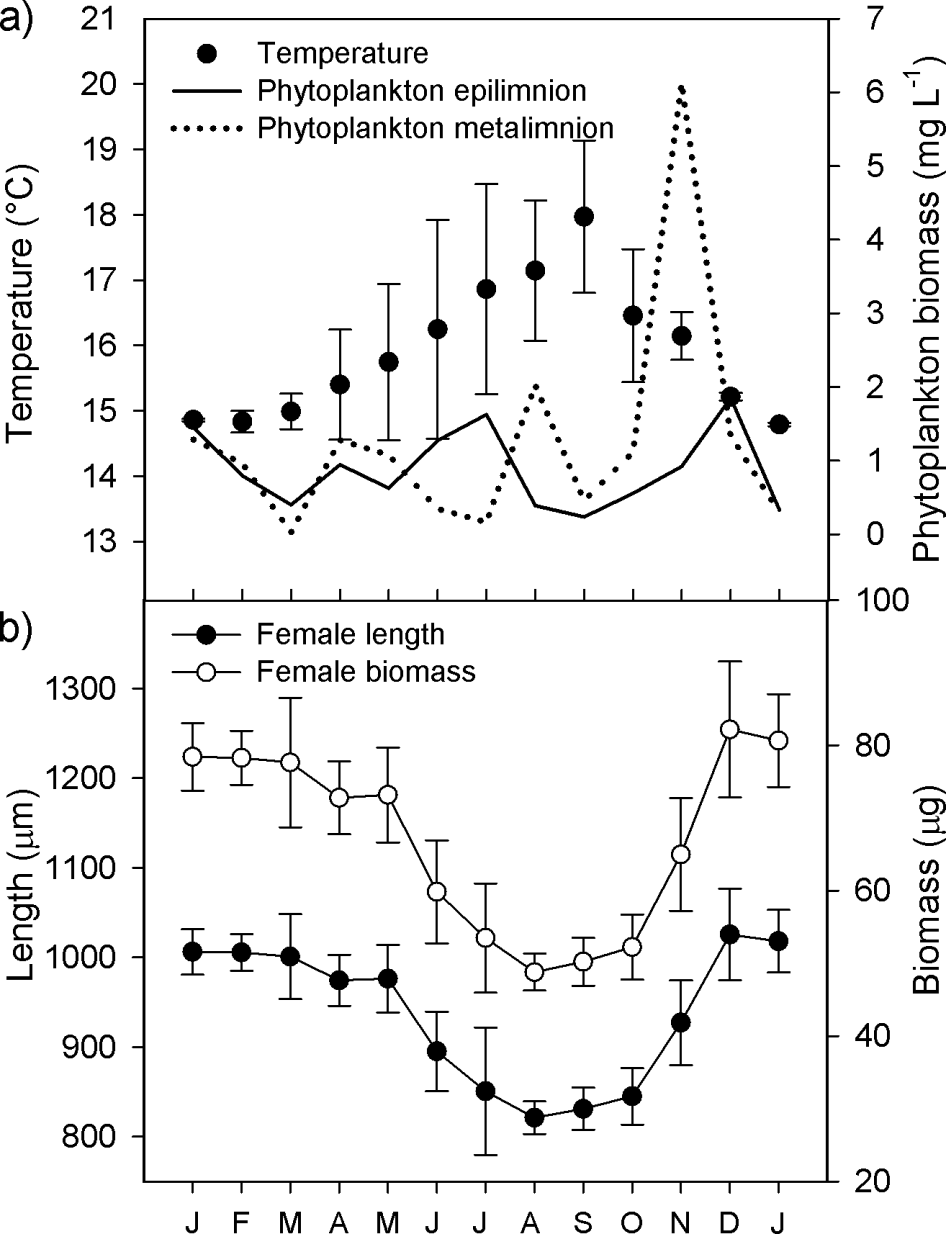
Temperature, phytoplankton biomass and adult female size of *Leptodiaptomus garciai* in Lake Alchichica. a) Average temperature of the oxygenated zone of the water column, and phytoplankton biomass at two depths, from January 2008 to January 2009. The averaged values of temperature correspond to the whole water column in the winter mixing and to the epilimnion and the metalimnion during the summer stratification. b) Adult female size of *Leptodiaptomus garciai* expressed as total length (cephalothorax plus urosome excluding the furcal rami) and wet weight. Vertical bars indicate ± one standard deviation.

Water column temperature fluctuated along the study period from 14.7°C (winter mixing and hypolimnion) to 19.7°C (surface in June). Average temperature in the oxygenated zone of the water column (epilimnion + metalimnion), where copepods thrive, increased slowly from a minimum in January (14.9 ± 0.1°C) to a peak in September (18.1 ± 1.2°C) (Fig 2A). Thus, the temperature annual range (TAR) was 3.3°C. Phytoplankton biomass followed the typical dynamics of Lake Alchichica [26]: it was homogeneously distributed during the mixing of the water column (1.4 ± 0.1 mg L^-1^ in January 2008) but as the lake stratified and the DCM developed it differed markedly between epilimnion and metalimnion (0.9 and 6.1 mg L^-1^, respectively, in November) (Fig 2A). In the oxygenated layer, the lowest mean phytoplankton biomass was observed in March (0.2 ± 0.3 mg L^-1^) and the highest in November (3.5 ± 3.6 mg L^-1^). Regarding the specific composition, the diatom *Cyclotella alchichicana* was the main component of the phytoplankton: during the mixing of the water column formed a bloom and afterwards it also dominated the metalimnetic DCM. At the beginning of spring, in March, the scarce phytoplankton biomass was composed by *C*. *choctawhatcheeana* plus decaying cells of *C*. *alchichicana*. During the stratification, at the epilimnion the most abundant species were successively the filamentous cyanobacteria *Nodularia spumigena* (May to June), and the diatoms *C*. *choctawhatcheeana* (August to October) and *Chaetoceros elmorei* (November and December).

From January to March 2008 and from December 2008 to January 2009, the average length (excluding furcal segment) and individual biomass of ovigerous females were high and relatively constant (1001 ± 47 μm to 1025 ± 51 μm; 77.6 ± 8.9 μg to 82.2 ± 9.3 μg, respectively). From April to November, length and biomass described a U-shaped curve, reaching the smallest value in August, when the means were 821 ± 18 μm and 48.8 ± 2.5 μg, respectively (Fig 2B).

**Fig 3:**
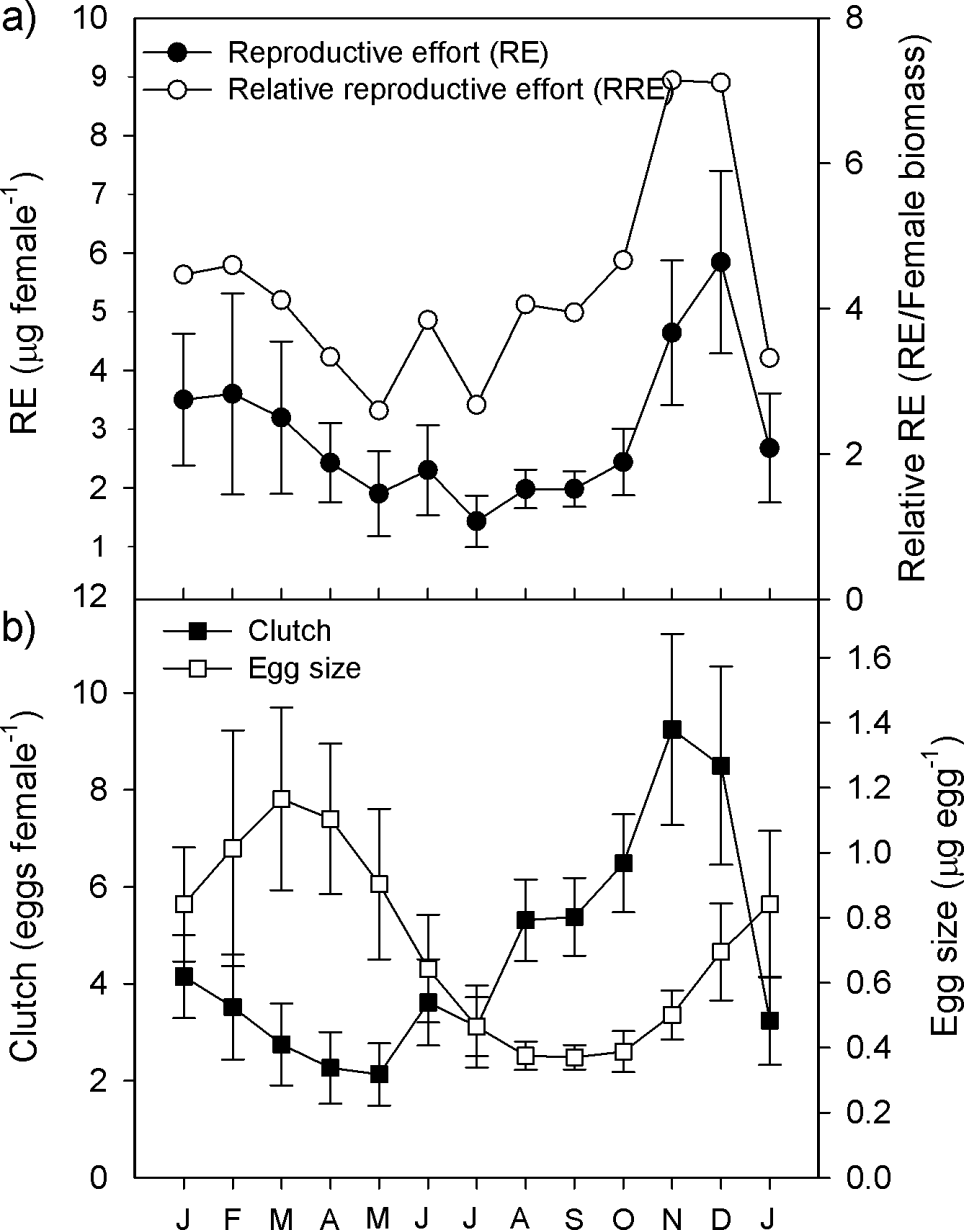
Temporal dynamics of life-history traits of *L*. *garciai* in Lake Alchichica. a) Average reproductive effort, i.e. the absolute quantity of biomass a female invests per clutch; and relative reproductive effort, the proportion of the female biomass invested per clutch. b) Average number of eggs per sac and average size of those eggs. Vertical bars indicate ± one standard deviation.

The curve of reproductive effort (Fig 3A) decreased steadily from January to July (minimum 1.4 ± 0.4 μg female^-1^) and then increased to a peak in December (5.8 ± 0.2 μg female^-1^). Reproductive effort was significantly correlated to female biomass (see below), so to remove the effect of female size and assess the effect of the environment we calculated the relative reproductive effort (RRE = RE*100/female biomass); this was lowest in May and July (2.6 ± 1.3%) and highest in November and December (7.1 ± 2.1%) (Fig 3A).

Clutch size and egg biomass also changed throughout the year (Fig 3B), the two describing sigmoid, approximately opposing, curves. Mean clutch size was lowest in May with 2.1 ± 0.6 eggs female^-1^, and increased steadily up to a maximum in November with 9.3 ± 2.0 eggs female^-1^. Mean individual egg biomass was highest in March (1.17 ± 0.28 μg) and lowest in August and September (0.37 ± 0.04 μg).

**Fig 4:**
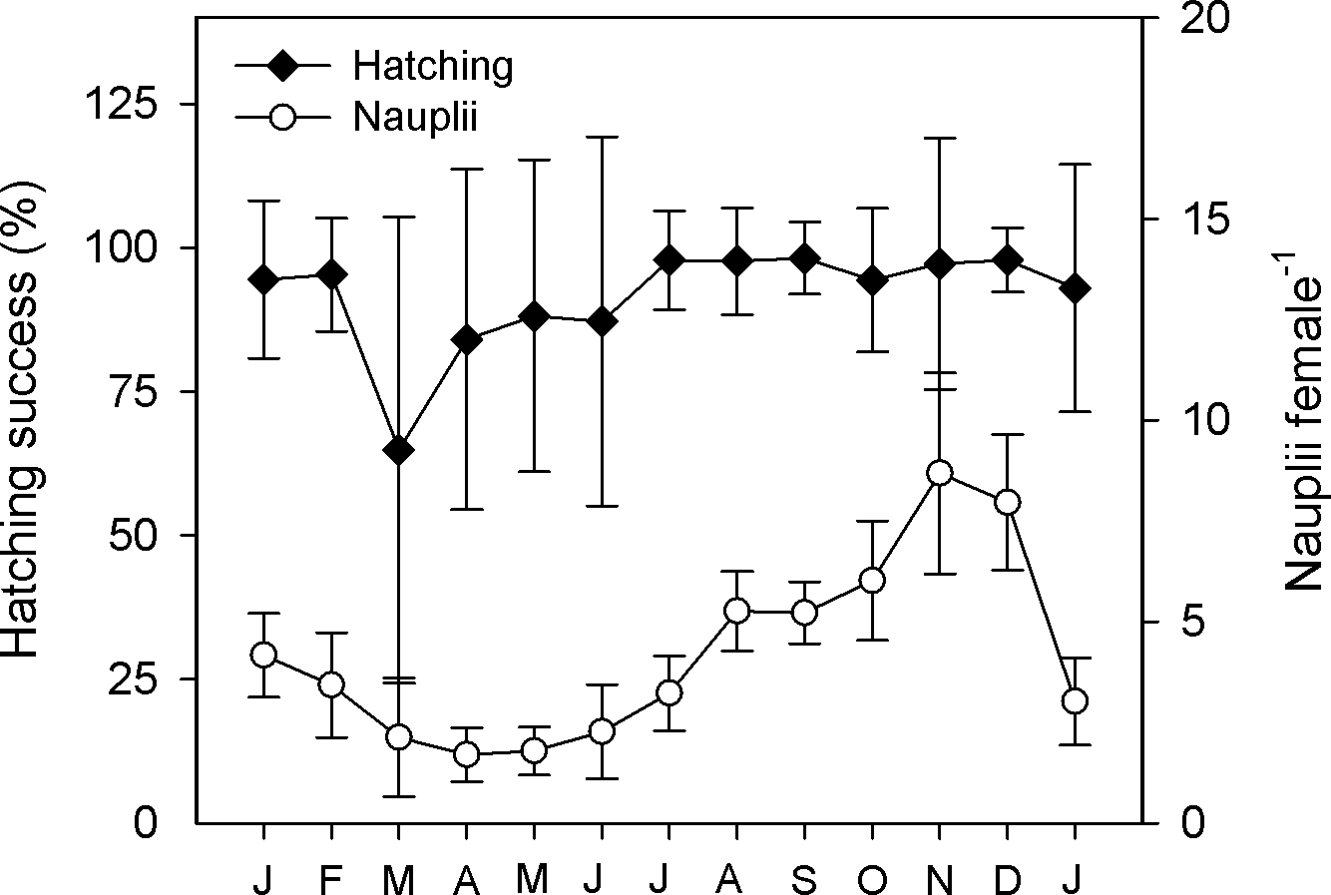
Temporal course of life-history traits of *L*. *garciai* in Lake Alchichica. Average proportion of eggs that hatched from a given egg sac and the average number of nauplii produced for each female. Vertical bar show ± one standard deviation.

Even though mean hatching success was usually above 90% (Fig 4), in spring (at the beginning of the stratification period), hatching decreased to a minimum of 65 ± 41% in March. The width of the standard error points to a high variability of hatching success among females in this period. Finally, the combination of clutch size and hatching success produced a sigmoid curve for the number of nauplii per female (Fig 4), with the lowest values in April and May (1.7 ± 0.7 and 1.8 ± 0.6 nauplii female^-1^) and the highest in November (8.7 ± 2.5 nauplii female^-1^).

**Fig 5:**
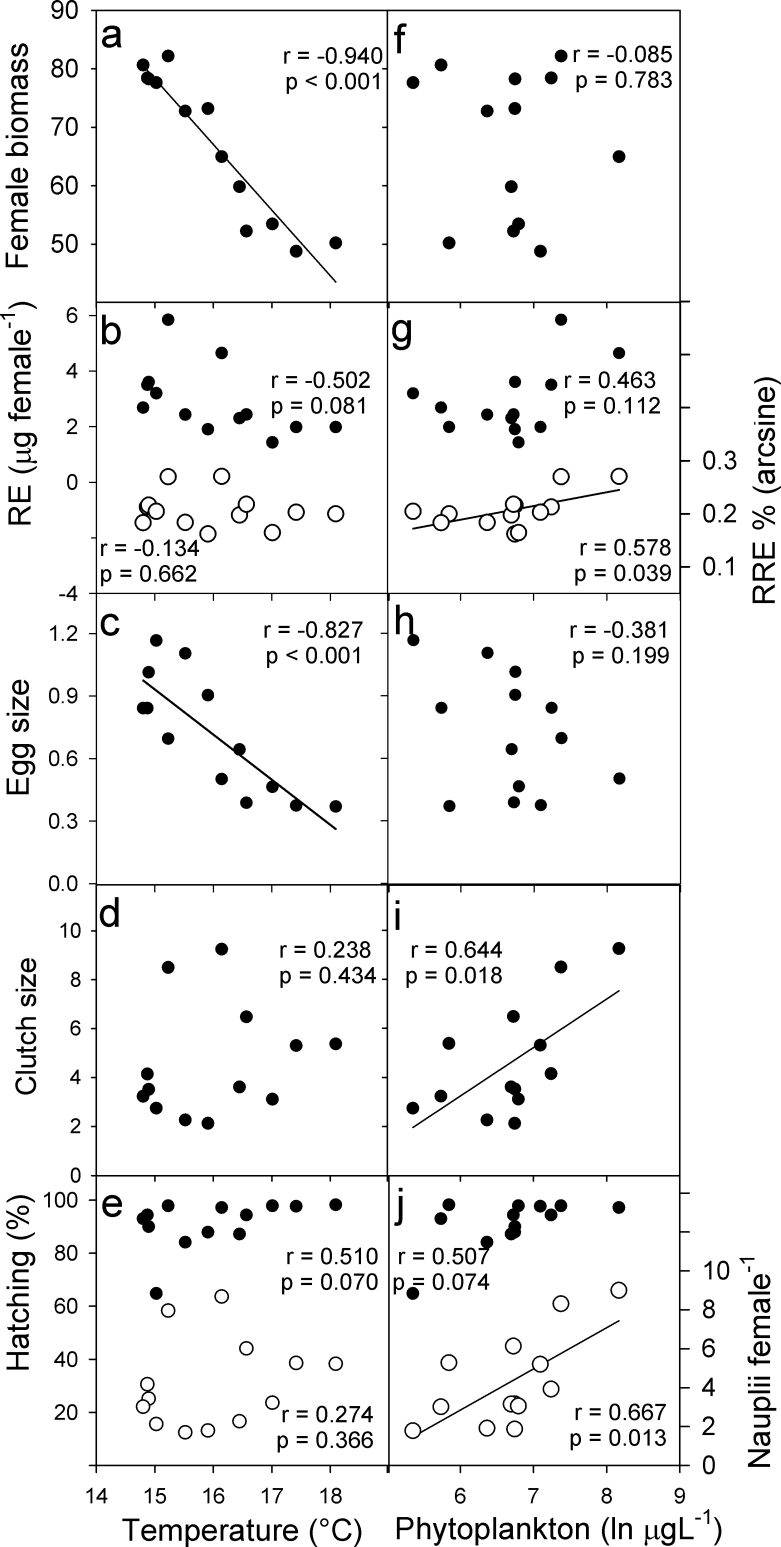
Dispersion plots of the average values of life-history traits of female *L*. *garciai* vs. temperature and food availability in Lake Alchichica. Probability and *r* coefficient values correspond to Pearson’s correlation tests; a significant correlation between the variability of a trait vs. temperature or phytoplankton is considered evidence of phenotypic plasticity to that factor.

The temperature of the water column was correlated significantly and negatively only with female length, female biomass and egg size (*p* < 0.001 in all instances; Fig 5A and 5C). Thus, the observed variability of these traits qualifies as phenotypic plasticity to temperature. Overall, considering maximum and minimum mean values, the Phenotypic Plasticity Index (PPI) was 41% for adult female biomass and 20% for female length, and was 68% for the individual egg biomass. The total phytoplankton biomass was positively correlated (*p* ≤ 0.05) to RRE, clutch size, and nauplii per female (Fig 5G, 5I and 5J); therefore, according to this criterion, these reproductive variables are plastic regarding resource availability, but egg size and female biomass are not. Overall, the PPI was 64% for the relative reproductive effort, 77% for clutch size, and 80% for nauplii per female. The absolute RE and hatching success were the only variables without evidence of variation associated with an environmental factor. The general additive models testing the simultaneous influence of phytoplankton and temperature on life-history traits gave significant effects for both environmental variables in absolute RE and in egg size. For RE, temperature had a negative effect, and phytoplankton was positive, whereas for egg size both variables had negative effect. For the remaining copepod traits, the explicative variable was only one, the same as for the Spearman or Pearson correlations (see S1 Table).

**Fig 6:**
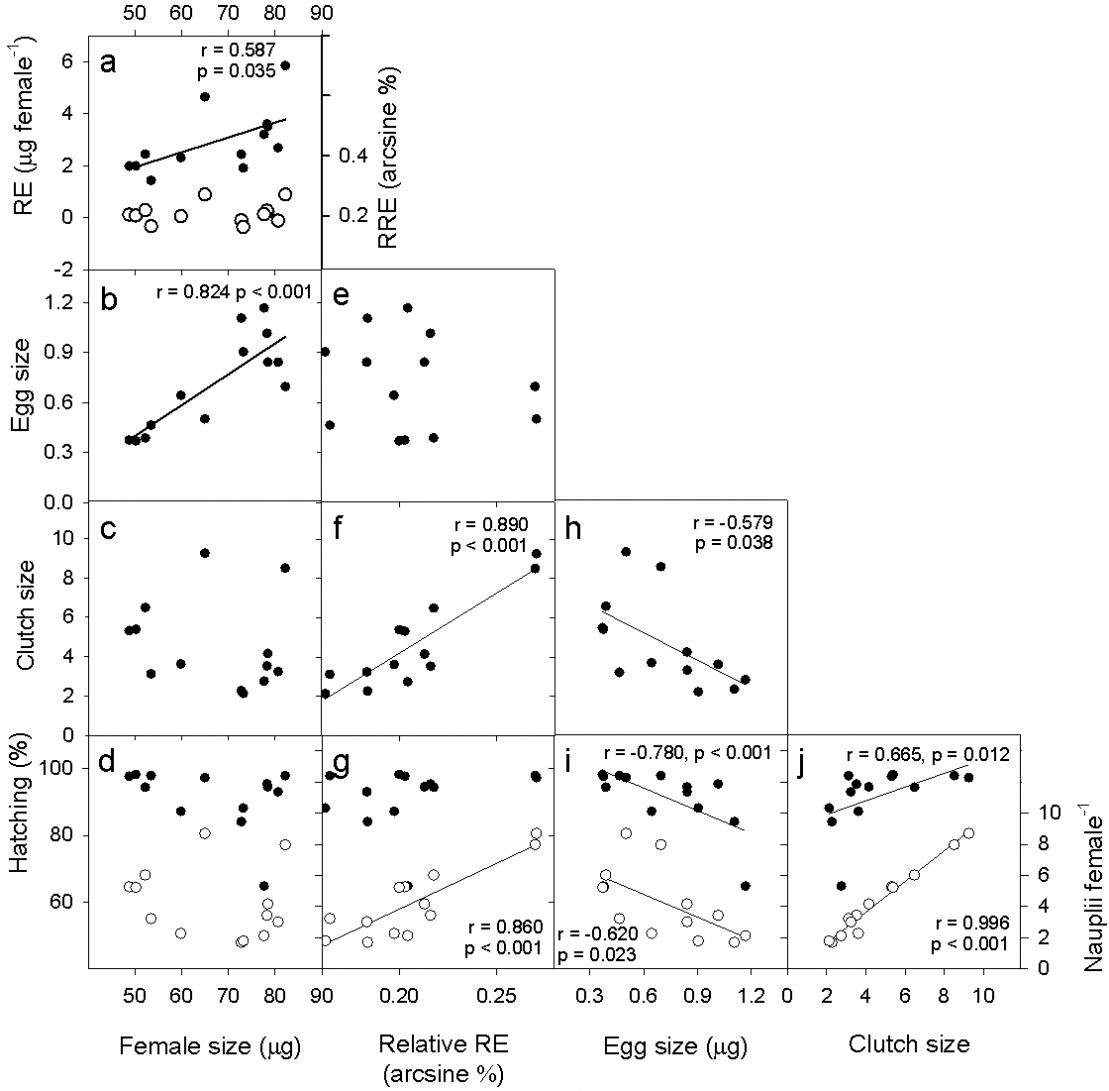
Relationships among the life-history traits of *L*. *garciai* in Lake Alchichica. Each point represents the population average at a given month. Probability and *r* coefficient values correspond to Pearson’s correlation tests; a significant negative correlation between two variables shows a potential functional trade-off. Open circles correspond to the left axis. RE, reproductive effort; RRE, relative reproductive effort (see text for further details).

Regarding relationships among life-history traits, we found that female size affected positively the RE (Fig 6A), i.e., the absolute biomass that females invested per reproductive event, but not the proportion of their own biomass invested in reproduction (RRE), which remained constant. Additionally, female size was positively correlated with egg size (Fig 6B). Interestingly, the variation in RRE was related to clutch size and nauplii per female (Fig 6F and 6G). The likely trade-offs were associated with egg size, which showed negative relationships with clutch size, hatching success and nauplii per female (Fig 6H and 6I). Finally, clutch size was associated positively with hatching success and nauplii per female (Fig 6J).

**Fig 7:**
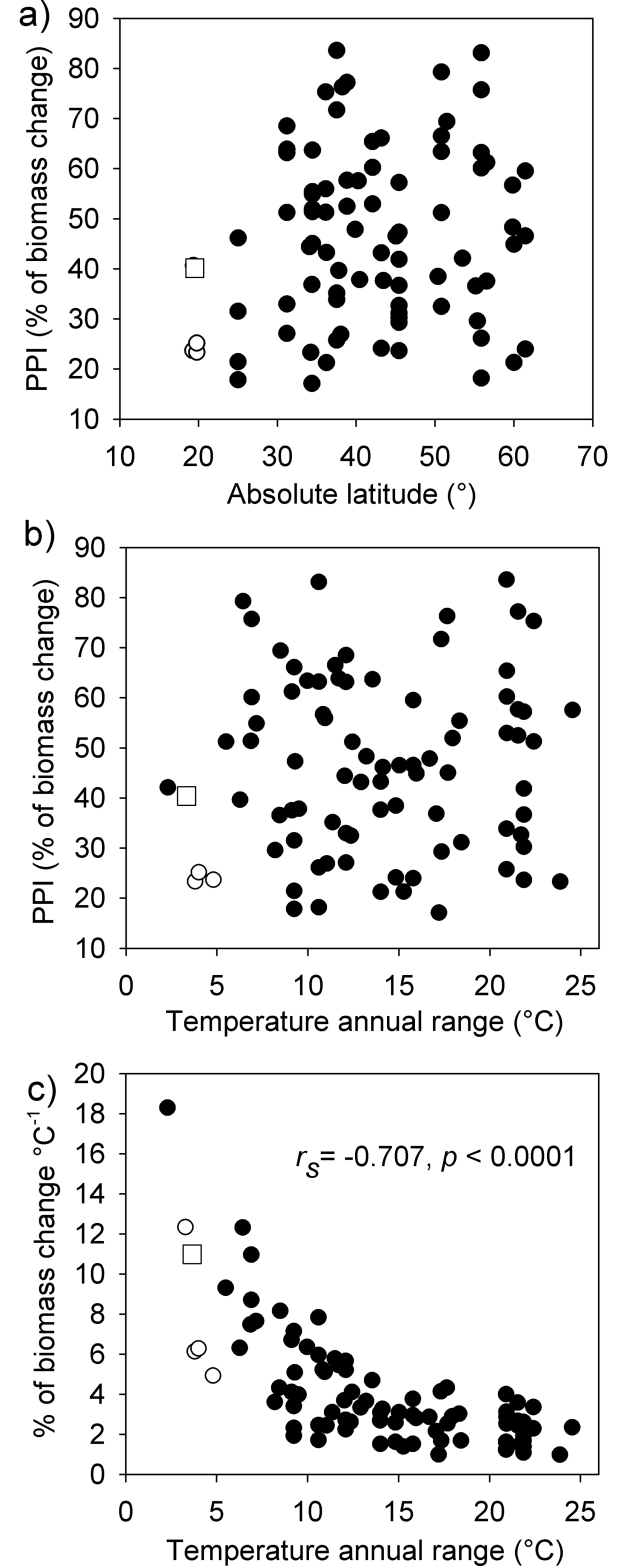
Phenotypic plasticity of female weight across a latitudinal range. a) Dispersion plot of the Phenotypic Plasticity Index (PPI) for adult female weight of 85 copepod populations (marine and lacustrine species) vs. the absolute latitude of their respective locations. b) Dispersion plot of the PPI for adult female weight of the same copepod populations vs. the TAR of their respective locations. c) Dispersion plot of the intensity of phenotypic expressed as % of change in biomass per °C (PPI/°C) vs. TAR, for the same populations. Open square: *L*. *garciai*; open circles: tropical populations; closed circles: temperate populations. Data were obtained from several sources, see details in Methods.

To evaluate the phenotypic plasticity of size of *L*. *garciai* within a global perspective, we calculated the PPI of female dry weight from the database of Horne *et al*. [21], which includes lacustrine and marine species, plus data from other papers reporting significant effects of temperature on copepod adult size (Fig 7A). Data from subtropical and temperate locations (between 25.0°N and 61.5°N) involving 80 populations outweigh by far the information from 5 populations from tropical latitudes. At tropical latitudes, the temperature annual range was narrow, between 3.2° and 5.0°C, with an average of 4.0 ± 0.8°C, whereas in temperate environments the temperature annual range ranged from 2.3°C in Lake Baikal, Russia, to 24.5°C in Lake Aquatina, Italy, with an average of 14.2 ± 5.3°C. A Mann-Whitney Rank Sum Test showed a significant difference in the median values between tropical and temperate locations (U statistic = 4.000, *p* = 0.001). However, we did not find a significant correlation between temperature annual range or seasonality and latitude (Spearman Rank Order correlation, *p* = 0.786, and *p* = 0.968, respectively; *n* = 85). In contrast, latitude was correlated negatively and significantly with the temperature of the coldest and the warmest months (*r* = − 0.622, *p* < 0.001 and *r* = −0.722, *p* <0.001, respectively). Accordingly, the mean minimum temperature in tropical locations was 22.2 ± 5.0°C, and 7.2 ± 5.2°C in temperate environments, whereas the mean maximum temperature was 26.2 ± 5.4°C in the tropical sites, and 21.6 ± 5.6°C in the temperate ones.

The absolute plasticity (PPI) of populations ranged between 16% (*Sinocalanus tennelus*) and 84% (*Ditrichocorycaeus affinis*), whereas the global average was 46% ± 18%. There was a significant positive correlation between the PPI and the mean body size of the distinct populations (Spearman Rank Order Correlation, *r* = 0.338, *p* = 0.002, *n* = 85), but not with species’ maximum under conventional analysis (*n* = 50) or with phylogenetically informed contrasts (number of valid contrasts = 39). In tropical populations the average PPI was 30 ± 8%, whereas in temperate organisms it was 50% ± 18%; this difference was statistically significant (U statistic = 57.0, *p* = 0.031). However, according to conventional statistical correlations performed with populations and species’ maximum (*n* = 50), as well as the phylogenetically informed analysis, the absolute PPI was not related to latitude, TAR or to seasonality (Table 1); for example, the highest PPIs, 84% (*D*. *affinis*) and 83% (*Pseudocalanus minutus*), occurred at temperature intervals of 21° and 11°C, respectively. In contrast, the conventional correlation analysis -at both population and species level- indicated a negative association of the PPI to the temperature of the warmest month, whereas the phylogenetically informed contrasts showed a marginally significant relationship of PPI only with the temperature of the coldest month.

**Table 1:** Correlation coefficients and associated probabilities for the relationship between the index of phenotypic plasticity (PPI) for adult female size and latitude and thermal variables.

	Populations (*n* = 85)	Species’ maximum (*n* = 50)	PICs of species’ maximum (*n* = 39)
Latitude	*r* = 0.139 (*p* = 0. 204)	*r* = 0.104 (*p* = 0.470)	*r* = 0.190 (*p* = 0.240)
*T*_min_	*r* = − 0.150 (*p* = 0.170)	*r* = −0.225 (*p* = 0.116)	*r* = 0.288 (*p* = 0.071)
*T*_max_	*r* = −0.222 (*p* = 0.041)	*r* = −0.300 (*p* = 0.035)	*r* = 0.160 (*p* = 0.324)
TAR	*r* = 0.049 (*p* = 0.657)	*r* = 0.031 (*p* = 0.831)	*r* = 0.174 (*p* = 0.284)
TS	*r* = 0.018 (*p* = 0.869)	*r* = 0.098 (*p* = 0.499)	*r* = 0.211 (*p* = 0.192)
% of change *vs* TAR	*r* = −0.707 (*p* < 0.001)	*r* = − 0.747 (p < 0.001)	*r* = −0.656 (p < 0.001)

For populations and species’ maximum, results correspond to Spearman Rank-Order correlations, the right column shows the results of the phylogenetically informed contrasts (PICs) for species’ maximum; for PICs *n* refers to the number of valid contrasts. *T*_min_ = Temperature of the coldest month, *T*_max_ = Temperature of the warmest month, TAR = Temperature annual range (*T*_max_−*T*_min_), TS = Temperature seasonality (standard deviation of the mean monthly temperature).

The intensity of phenotypic plasticity per °C ranged from 1.0% of biomass °C^-1^ (*Oithona davisae*) to 18.3% of biomass °C^-1^ (*Epischura baicalensis*), whereas for *L*. *garciai* was 12.7% of biomass °C^-1^ (Fig 7B). In this case, the average was significantly higher in tropical populations (7.5 ± 3.6% of biomass °C^-1^) than in temperate ones (4.0 ± 2.8% of biomass °C^-1^) (U statistic = 48.0, *p* = 0.019). Finally, there was a significant negative correlation between PPI°C^-1^ and TAR (Fig 7B); this relationship was observed for population and species datasets analysed by conventional statistics, as well as considering their phylogeny (Table 1).

## Discussion

The original data presented in this study provide evidence of the plasticity observed for an endemic species which up to date has been found only in Lake Alchichica [36]. The hydrological cycles and the accompanying oscillations in the abiotic and biotic variables of Lake Alchichica have been widely documented for the last 20 years [30, 61], so even though we analysed data from only one year, we judge our results reflect the response of the copepod population facing an average year. However, considering the subtle but apparently steady increase in water temperature observed the last years, it would be desirable to extend the survey to a longer period, as well as to include other species from neighbour waterbodies (in this lake, *L*. *garciai* is the only pelagic copepod that dominates the planktonic community along the year). In addition to temperature and food, there are several environmental factors that fluctuate seasonally in Lake Alchichica and which may affect behaviour and fitness of copepods. Temperature fluctuation throughout the year produces the alternation between mixing and stratification of the water column, resulting in changes in nutrient availability, transparency, depth of the oxygenated zone, etc., affecting also the dynamics of potential food resources, competitors and predators [26, 27]. Even tough according to our results, temperature was the most important driver of size of adult females, the effect of food quantity and quality on the individual development cannot be discarded. On the other hand, the presence of visual predators that selectively feed on larger prey could result in the elimination of larger copepod phenotypes [62], reinforcing the effect of higher temperatures during the stratification period [27].

### Phenotypic plasticity in *L*. *garciai*

As expected, the variability of temperature in Lake Alchichica was very restricted, but we could verify that copepods possess phenotypic plasticity in response to temperature in an important life history-trait: adult female size. Female size was not related to phytoplankton biomass, which varied 17-fold along the year. However, three life-history traits did respond significantly to food availability: relative reproductive effort, clutch size and number of nauplii per female. The simultaneous effect of temperature and phytoplankton was observed on egg size and reproductive effect, which were also related to female size.

Average female size oscillated regularly through time in Lake Alchichica and showed a clear pattern of thermal plasticity. The negative correlation of female size with environmental temperature is clearly consistent with previously reported results from continental and marine copepod species from temperate environments [7,21], and with ectotherm animals in general [3]. In fact, the phenotypic plasticity index [PPI] for *L*. *garciai* adult female size was the highest among the tropical species, but very according to the global average. As phytoplankton availability did not exert a statistically significant effect on female size, we assume that temperature, not food availability, can be the most important driver in determining the size of adult copepods even in environments where the temperature annual range is under14°C, contradicting previous hypothesis [11]. Our results extend the scope of the conclusion of Horne *et al*. [21] that in most cases temperature has a negative relationship to adult size in copepods, in that it can be the most important driver of this trait not only in temperate but also in tropical environments.

Temperature -along with phytoplankton- was also negatively correlated with the absolute quantity of biomass that females invest in reproduction (RE) and egg size, and both traits were explained by female size. Thus, at lower temperatures, females were larger, invested more in reproduction and produced larger eggs. Temperature affects the offspring size of ectotherms in two ways: directly, by regulating the embryo development, and indirectly by influencing the maternal body size. However, the relative importance of each element is not always clear; it can differ between species and should be better assessed experimentally [63, 64]. Regarding the role of phytoplankton on egg size variation, at lower food availability, eggs were larger. This result is in accordance with the trade-off between egg size and clutch size discussed below. Due to the complex interplay of environmental factors and trade-offs involved in the temporal variation of egg size, a pattern of phenotypic plasticity cannot be elucidated here, we only point out that the temporal variation of egg size of *L*. *garciai* is appreciably wider (68%) than the highest value for this trait hitherto reported for a copepod: 47% for *Pseudodiaptomus marinus*, which lives in an environment with a temperature variation of 19.3°C [10].

The narrow variation of RE observed in *L*. *garciai* might be adaptive, since there should be an upper limit to the additional weight females can carry without interfering significantly with their own fitness. Ovigerous females are more exposed to visual predation than non-ovigerous females because they do not migrate vertically [65] or do it at lower rates, and are more easily targeted by fish, so any increase in the size of the egg mass could compromise the survival of the female. However, the RE and the relative reproductive effort (RRE), i.e. the proportion of female biomass invested in every reproductive event, as well as the clutch size, showed a positive response to phytoplankton availability; this suggests that once an individual has reached adult size (copepods have a determinate growth), surplus energy is diverted into reproduction. As in other copepod species, *L*. *garciai* females convert the assimilated food into reproduction by adjusting the number of eggs laid per clutch, and thereby increasing their reproductive success [66].

The amplitude of the phenotypic plasticity of clutch size observed in this species (77%) is similar to the 80% observed in *Euterpina acutifrons* [67], which was also positively related to food availability. The positive effects of food quantity and quality on clutch size have been observed in many other copepod species [35], and significant relationships with temperature [10,19] and female size [68] have also been observed.

The negative correlation between egg size and clutch size satisfies the statistical condition of a functional trade-off; the reproductive effort is divided between the number and the size of the offspring. Life-history theory postulates that the fitness of the progeny is directly related to its size at birth; for example, larger eggs store more energy and can provide the larvae with higher chances of survival at the beginning of life if food is scarce [69], and these larvae can usually acquire resources more efficiently [39]. In some egg-carrying zooplankters production of few but large eggs under low food conditions increases the reproductive success of individuals, because large offspring show higher fitness; in contrast, when food is abundant the best strategy could be to increase the number of eggs, even if they are small and have reduced energy reserves [67]. This suggests an adaptive function for this reproductive allocation. However, for this explanation to be valid, it is necessary that both egg size and number are regulated by food availability. In the case of *L*. *garciai* the allocation “decisions” did not depend on a single factor, since egg number was influenced by resources, whereas egg size was also constrained by temperature (directly or indirectly), resulting in sigmoidal trajectories that are not always opposite but slightly “out of phase”. For example, there were periods during which both size and number of eggs decreased (from March to May) or both increased (from September to November). Moreover, the smallest eggs (August-October) were produced within clutches of intermediate size.

A further trait that can inform on the adaptiveness of the size and number of eggs is the hatching rate, which was high except for a period during spring when it decreased notably. The variability of hatching was not related to temperature or to food availability, but it was correlated positively to the number of eggs and negatively to the size of the eggs, because the largest eggs −which formed small clutches− had the lowest hatching rates. Hatching rate is the only measure of fitness that we have on offspring, but the result is contrary to the response observed by Guisande *et al*. [67], who concluded that the higher fitness of large eggs is due, at least in part, to their higher hatching rate. Ianora *et al*. [70] found that the question of a relationship between egg size and hatching success in copepods “remains largely unsolved”, in the face of conflicting reports of positive and negative relationships and even a complete absence of association; in two cases, low hatching rates of large eggs had been attributed to maternal effects due to a diet of low-quality phytoplankton (decaying cells), a mechanism that should be further investigated in Lake Alchichica, because the lowest hatching rates occurred when the winter bloom of diatoms was ending.

Integration of the present results shows that the largest eggs were present in small clutches, and tended to have low hatching rates, a combination that resulted in fewer offspring for each female. In contrast, the fitness of females increased with larger clutch sizes; even when eggs where smaller owing to thermal conditions, they had higher hatching rates. As the number of eggs depended on phytoplankton availability, the capacity of females to increase the number of eggs when food is abundant seems to be a great advantage (it would be adaptive) in an environment such as Lake Alchichica, where phytoplankton shows wide fluctuations. When food supply is low (at the beginning of spring), females divert fewer resources to reproduction, and perhaps increase the likelihood of their own survival; however, oocytes that do not receive enough nourishment are degraded, and the remaining eggs can have an inadequate content of lipids and yolk [71]. The deficient biochemical quality of eggs produced in limiting feeding conditions can in turn be the cause of the low hatching rate. The size of eggs in that case would be just a consequence of the environmental temperature in which mothers and embryos developed, and would be unrelated to the quality of the offspring and without adaptive value. Thus, the way resources are allocated between the offspring is probably not an optimized strategy, but a fortuitous response to a given combination of environmental factors, and the existence of a statistical trade-off can be merely a coincidence due to the trajectories of the driving factors of each trait. However, the lack of adaptive value of the variability of egg size in this species is a hypothesis that certainly needs further testing, comparing the survival and development of nauplii born from eggs produced under different conditions of temperature and food supply.

Although the inverse relationship between temperature and size in copepods has been considered adaptive by some authors [7,72], the scarcity of mechanistic studies to explain this pattern, and the contradictory evidence in some cases, precludes any generalisation. The size at which organisms stop growing and start reproducing has consequences for their life histories and individual fitness [39]. For example, large females can feed on a wider range of food items, but small females start to reproduce sooner. In our study, large *L*. *garciai* females could allocate a larger quantity of biomass to reproduction, and their offspring were larger, traits usually interpreted as advantageous [39]. However, those large eggs showed a low hatching success, decreasing significantly the number of larvae produced by each copula, compared with small females. From this point of view, the best phenotype for females seems to be the small one, and consequently the thermal plasticity of females would be non-adaptive. However, as mentioned above, it is necessary to gather experimental evidence on the further performance of the descendants of both small and large females, because higher hatching rates may not imply higher survival rates of individuals in the long term.

Finally, we are aware that other factors, as visual predation, can affect the mean size of the adult copepods. The activity of visual predators that feed selectively on larger prey could result in the elimination of larger copepod phenotypes [62]. This event is more likely to occur during the stratification periods, when copepods are not able to migrate downwards to avoid visual predation due to the anoxic conditions of the hypolimnion, as we described in another paper [27]. Thus, the elimination of the larger females would reinforce the effect of the highest temperatures to render the lowest mean body size.

### Thermal plasticity in the latitudinal gradient

According to the Climate Variability Hypothesis, it was expected that *L*. *garciai*, which inhabits a tropical lake with narrow thermal variability (TAR), would show limited or no thermal plasticity. However, it exhibited a consistent and significant negative trend of change in adult size and egg size according to seasonal oscillations in water temperature, as has frequently been described in temperate environments but not in the tropics. The absolute range of thermal plasticity in the female size of *L*. *garciai* appears in the middle of the data cloud of thermal plasticity (PPI) vs. latitude or TAR; this verifies thermal plasticity across the whole range of latitudes where data on copepods exist. Considering the latitudinal and seasonal distribution of the absolute plasticity in the whole dataset, we infer that the amplitude of the thermal plasticity is not clearly related to the range of temperature variation. However, a closer look at the analysed dataset revealed that in these aquatic environments, latitude is not an explicative variable of the thermal annual range (*r* = −0.030, *p* = 0.786, *n* = 85) neither of temperature seasonality (*r* = −0.004, *p* = 0.968, *n* = 85). This lack of relationship could be due to local factors like altitude, size, depth and morphology of lakes or distance to the coast, oceanic currents, upwellings or salinity gradients in the sea, just to mention a few [8]. Lake Alchichica is a good example of how local factors can affect latitudinal predictions on thermal limits and variability, because it displays the narrow temperature variability expected for a tropical lake [73], but both maximum and minimum temperatures are comparatively low due to its altitude. On the other hand, the climatic variability hypothesis (CVH) was proposed to explain the amplitude of tolerances required by terrestrial animals to withstand the climatic fluctuation experienced in a given location. Aquatic arthropods show stronger species-specific temperature-size responses than terrestrial species, probably because changes in water temperature generate more challenging conditions for animals than in terrestrial environments: increases in temperature demand higher metabolic rates, causing simultaneously a lower oxygen availability [74]. However, this pattern does not explain why the amplitude of the plasticity is not proportional to the thermal variability in an interspecific comparison. Accordingly, we did not find support for the Climatic Variability Hypothesis for phenotypic plasticity in this group of aquatic organisms.

One the other hand, we found that water temperature is not insensitive to latitudinal influence, because latitude was negatively related to the temperature of both the coldest and the warmest month (*r* = −0.622, *p* < 0.001, *n* = 85; *r* = − 0.722, *p* < 0.001, *n* = 85, respectively), implying that at lower latitudes, maximum and minimum temperatures are higher than in the temperate zone. Regarding the latitudinal variation of phenotypic plasticity of female size, this is a meaningful result, because according to conventional statistics, the PPI was explained by the temperature of the warmest month (negative) although with low coefficients, whereas the phylogenetically informed contrasts showed a marginal positive effect of the temperature of the coldest month. Thus, the phenotypic plasticity of copepod adult size could be associated to latitude, but due to the maximum and/or minimum temperatures, not to the range of temperature. However, the statistical evidence of this relationship is weak, and should be further investigated with a mechanistic point of view in mind.

In contrast, an interesting pattern was observed in the proportion of change in mass per °C, which was higher in populations experiencing low temperature variation. We found that the phenotypic plasticity was more intense at narrower temperature intervals, denoting that though the absolute plasticity may be similar across the latitudinal gradient, there should be important differences in the sensitivity of organisms to temperature change. Evidently, it is essential to gather more relevant data from tropical species, both lacustrine and marine, to verify this pattern and to explain mechanisms and their adaptive significance.

## Supporting information

S1 Dataset Original data of temperature and dissolved oxygen of Lake Alchichica and life-history traits of *L*. *garciai*. (XLSX)

S1 Table Summary of GAM models of life-history traits of *L*. *garciai* depending on temperature (Temp) and phytoplankton (Phyto) assuming a smooth function (s). (DOC)
