# Supplementary material for: Phenotypic plasticity of life-history traits of a calanoid copepod in a tropical lake: Is the magnitude of thermal plasticity related to thermal variability?
Source: PLoS One. 2018 Apr 30;13(4):e0196496. doi: 10.1371/journal.pone.0196496 (PMC5927456; doi:10.1371/journal.pone.0196496)
Supplement: S1 Table — (DOC) [file pone.0196496.s002.doc]

**Supplementary Material**

**General additive models**

We evaluated effects of both environmental factors (i.e., temperature and phytoplankton) on each of the life-history traits of copepods using generalized additive models (GAMs) fit in R v. 3.4.2. [1] using the *gam* package [2,3]. The effect of temperature and phytoplankton on the monthly average of every trait was analyzed using a Gaussian error distribution and the identity link function. Temperature (Temp) and phytoplankton biomass (Phyto) were the additive factors, with the smoothing function (s) tested alternatively on the variables on full, LM or partial models. The selection of the best fitted model was based on the Akaike’s Information Criterion and Deviance, tested with the *anova* function, assessed by a χ^2^-statistic [4]. If one of the two variables in the best fitted model showed an individual *p* > 0.05 it was considered non-significant (Ns), whereas the remaining variable was considered the explicative one (hypothesis testing about model terms; [4]).

**S1 Table.** **Summary of GAM models of life-history traits of *L. garciai* depending on temperature (Temp) and phytoplankton (Phyto) assuming a smooth function (s)**.

|  | **Factor (*p*-values)** | |  | **Model parameters and comparison** | | | | |
| --- | --- | --- | --- | --- | --- | --- | --- | --- |
| **Model** | **Temp** | **Phyto** |  | **r^2^** | **Deviance explained** | **GCV** | **AIC** | ***p*-values** |
| **Female size** |  |  |  |  |  |  |  |  |
| LM | <0.001 | Ns |  | 0.875 | 89.6 | 25.64 | 80.24 |  |
| s(Temp)+Phyto | <0.001 | Ns |  | 0.936 | 96.0 | 17.23 | 72.75 | 0.009 |
| Temp + s(Phyto) | <0.001 | Ns |  | 0.875 | 89.6 | 25.64 | 80.25 | 0.006 |
| s(Temp) | <0.001 | − |  | 0.944 | **96.0** | 13.77 | **70.86** | <0.001 |
| s(Phyto) | − | Ns |  | -0.090 | 0.03 | 204.02 | 107.69 | <0.001 |
| **Clutch size** |  |  |  |  |  |  |  |  |
| LM | Ns | 0.004 |  | 0.511 | 59.3 | 3.30 | 53.71 |  |
| s(Temp)+Phyto | Ns | 0.004 |  | 0.511 | 59.3 | 3.30 | 53.71 | <0.001 |
| Temp + s(Phyto) | Ns | 0.018 |  | 0.622 | **75.2** | 3.26 | **51.49** | 0.13 |
| s(Temp) | Ns | − |  | -0.046 | 4.12 | 6.48 | 62.85 | 0.31 |
| s(Phyto) | − | 0.003 |  | 0.513 | 55.4 | 3.01 | 52.89 | 0.28 |
| **Egg size** |  |  |  |  |  |  |  |  |
| LM | <0.001 | Ns |  | 0.717 | 76.4 | 0.03 | -7.64 |  |
| s(Temp)+Phyto | 0.002 | 0.04 |  | 0.785 | **85.6** | 0.03 | **-10.07** | 0.14 |
| Temp + s(Phyto) | <0.001 | 0.07 |  | 0.718 | 76.6 | 0.03 | -7.67 | 0.14 |
| s(Temp) | <0.001 | − |  | 0.636 | 66.6 | 0.04 | -5.13 | 0.02 |
| s(Phyto) | − | Ns |  | 0.020 | 10.2 | 0.09 | 7.73 | 0.16 |
| **ER** |  |  |  |  |  |  |  |  |
| LM | 0.021 | 0.014 |  | 0.543 | 61.9 | 0.906 | 36.78 |  |
| s(Temp)+Phyto | 0.004 | 0.016 |  | 0.935 | **98.5** | 0.455 | **9.41** | <0.001 |
| Temp + s(Phyto) | 0.010 | 0.042 |  | 0.810 | 89.5 | 0.571 | 26.79 | 0.002 |
| s(Temp) | 0.061 | − |  | 0.218 | 28.3 | 1.410 | 43.02 | <0.001 |
| s(Phyto) | − | 0.014 |  | 0.712 | 82.3 | 0.765 | 32.07 | <0.001 |
| **ERR** |  |  |  |  |  |  |  |  |
| LM | Ns | 0.005 |  | 0.490 | 57.5 | 1.327 | 41.75 |  |
| s(Temp)+Phyto | Ns | 0.004 |  | 0.504 | 59.6 | 1.320 | 41.54 | 0.13 |
| Temp + s(Phyto) | Ns | 0.020 |  | 0.609 | 74.5 | 1.300 | 39.45 | 0.13 |
| s(Temp) | Ns | − |  | -0.061 | 2.7 | 2.511 | 50.52 | 0.14 |
| s(Phyto) | − | 0.011 |  | 0.663 | **76.0** | 1.025 | **37.22** | 0.06 |
| **Hatching** |  |  |  |  |  |  |  |  |
| LM | Ns | Ns |  | 0.181 | 31.7 | 91.01 | 96.71 |  |
| s(Temp)+Phyto | Ns | Ns |  | 0.187 | 32.7 | 90.98 | 96.67 | 0.13 |
| Temp + s(Phyto) | Ns | Ns |  | 0.290 | **44.4** | 84.06 | **95.26** | 0.12 |
| s(Temp) | Ns | − |  | 0.072 | 14.9 | 93.76 | 97.58 | 0.08 |
| s(Phyto) | − | Ns |  | 0.182 | 28.8 | 86.96 | 96.37 | 0.13 |
| **Nauplii** |  |  |  |  |  |  |  |  |
| LM | Ns | 0.005 |  | 0.502 | 58.5 | 3.38 | 53.90 |  |
| s(Temp)+Phyto | Ns | 0.003 |  | 0.551 | 64.6 | 3.22 | 52.91 | 0.15 |
| Temp + s(Phyto) | Ns | 0.016 |  | 0.618 | **74.4** | 3.21 | **51.51** | 0.15 |
| s(Temp) | Ns | − |  | -0.040 | 4.7 | 6.41 | 62.70 | 0.001 |
| s(Phyto) | − | 0.004 |  | 0.499 | 54.0 | 3.09 | 53.22 | <0.001 |

The right column depicts *p*-values derived from ANOVA tests, assessed by a χ^2^-statistic [4] for significance testing of effects. Bold font indicates the minimum adequate model according to AIC and deviance. GCV: generalized cross validation score; AIC: Akaike’s Information Criterion. Ns: Not significant (*p* > 0.05).

**References**

1. R Development Core Team. R: a language and environment for statistical computing. version 3.4.2. 2017 Sep 28 [cited 2018 Apr 1]. R Foundation for Statistical Computing. Available from http://www.R-project.org/
2. Hastie TJ, Tibshirani RJ. Generalized additive models. New York: Chapman and Hall; 1990.
3. James G, Witten D, Hastie T, Tibshirani R. An introduction to statistical learning: with applications in R. New York: Springer; 2013.
4. Wood S. Generalized additive models: an introduction with R. 2nd ed. CRC Press; 2017.
